# *XMeis3* Is Necessary for Mesodermal *Hox* Gene Expression and Function

**DOI:** 10.1371/journal.pone.0018010

**Published:** 2011-03-28

**Authors:** Paul M. J. In der Rieden, Hans J. Jansen, Antony J. Durston

**Affiliations:** 1 Studiekring, Utrecht, Netherlands; 2 Sylvius Laboratory, Leiden, Netherlands; National University of Singapore, Singapore

## Abstract

Hox transcription factors provide positional information during patterning of the anteroposterior axis. Hox transcription factors can co-operatively bind with PBC-class co-factors, enhancing specificity and affinity for their appropriate binding sites. The nuclear localisation of these co-factors is regulated by the Meis-class of homeodomain proteins. During development of the zebrafish hindbrain, Meis3 has previously been shown to synergise with Hoxb1 in the autoregulation of *Hoxb1*. In Xenopus XMeis3 posteriorises the embryo upon ectopic expression. Recently, an early temporally collinear expression sequence of *Hox* genes was detected in *Xenopus* gastrula mesoderm (see intro. P3). There is evidence that this sequence sets up the embryo's later axial Hox expression pattern by time-space translation. We investigated whether XMeis3 is involved in regulation of this early mesodermal Hox gene expression. Here, we present evidence that XMeis3 is necessary for expression of *Hoxd1*, *Hoxb4* and *Hoxc6* in mesoderm during gastrulation. In addition, we show that XMeis3 function is necessary for the progression of gastrulation. Finally, we present evidence for synergy between XMeis3 and Hoxd1 in *Hoxd1* autoregulation in mesoderm during gastrulation.

## Introduction

During the development of most animal species studied, *Hox* transcription factors specify positional information along the anterior - posterior axis [1]–[5]. *Hox* genes comprise a subfamily of the homeobox containing gene family, and are organised in four clusters, each located on a different chromosome. The homeobox encodes a DNA binding motif called the homeodomain. A strict control of the expression and function of these *Hox* genes is essential.

It has been shown that Pbx family members, and their *Drosophila melanogaster* counterpart *Extradenticle* (Exd), function as cofactors for Hox proteins; they can enhance their binding specificity and affinity for specific target sequences in DNA [6]–[10]. Pbx/Exd family members are part of a particular subfamily of the homeodomain containing proteins, namely the TALE-class. This class is characterised by having a three amino acid loop extension between the first and second helices of the homeodomain [11]. It has been proposed that cooperative binding of Hox and Pbx/Exd proteins can lead to transactivation while binding of the individual factors leads to repression on the same promoter elements [12]. When Hox proteins bind to DNA cooperatively with a Pbx/Exd family member, the main protein-protein interaction consists of binding of the hexapeptide motif of the Hox protein to a pocket formed by the atypical homeodomain of PBC family members [13]–[15] This pocket is composed of the three amino acid loop extension of the PBC homeodomain, residues in the third helix of the homeodomain, and a residue in the C-terminal helix of PBC homeodomains [15]. The nuclear localisation of Pbx/Exd proteins is controlled by competing nuclear import and export signals [16]. When members of the Meis family, or their *Drosophila* counterpart *Homothorax* (Hth), also members of the TALE-class of homeodomain proteins, are present in the cytoplasm they can interact with Pbx/Exd family members in such a way that the nuclear export signal of the Pbx/Exd family member is shielded, resulting in a net influx of Pbx/Exd into the nucleus, sometimes influencing the function of Hox proteins present [9], [17], [18]. However, Pbx/Exd and Meis/Hth proteins are not used exclusively as cofactors for Hox proteins. The myogenic bHLH factors [19] and Engrailed [20] also depend on the activity of Pbx and Meis members for proper functioning.

For Hox paralog group 1 members, autoregulation dependent on Pbx/Exd and Meis/Hth has been shown in the neurectoderm of mouse embryos [21], [22], in *C. elegans* [23] and in endoderm of *Drosophila* embryos [17], [24]. Binding of Hox and Pbx family members to bipartite Hox-Pbx binding sites is essential for autoregulation [17], [21], [24], [25]. Meis proteins have been shown to be indispensable as mediators of this process [17], [24], [25].

In *Xenopus*, a member of the Meis family, *XMeis3*, is a posteriorising factor in the neurectoderm of *Xenopus laevis*, and is required for hindbrain patterning [26], [27]. Recent findings also show that neurectodermal *XMeis3* mediates the posteriorising action of *XWnt3A* in the developing CNS [28]. In zebrafish embryos, similar functions have been reported for Meis3 and other Meis family members [29]–[31]. Expression of *XMeis3* is reported as being initiated in a stripe in the neural plate of early-mid neurula embryos. During neurula and early-tailbud stages, expression is mainly localised to rhombomeres (r's) 2, 3, and 4, and the anterior spinal cord, while posterior rhombomeres show some ventral expression [26]. Expression of *XMeis3* overlaps with neurectodermal expression of *Hoxd1* (r4 and r5) [32], *Hoxb4* (r7, r8, and the anterior spinal cord) [33], and *Hoxc6* (anterior spinal cord) [34], [35]. These overlaps are consistent with the idea that *XMeis3* is involved in controlling the function of the Hox proteins with which it is co-expressed. These studies do, however, leave many questions unanswered. They pay little attention to when and where Meis cofactors actually interact with Hox proteins at different stages during the early AP patterning process. These details are likely to be crucial for understanding the mechanism at hand. Studies of vertebrate *Hox* expression and function have already delivered strong evidence that AP patterning depends on a specific early spatiotemporal sequence of *Hox* gene expression. Expression of each Hox gene is initiated in a specific mesodermal domain in the gastrula embryo and then undergoes an establishment phase during which this expression domain changes to a gene specific AP zone in axial mesoderm and the neural plate and finally a maintenance phase during which this AP zone is consolidated. This sequence is employed universally in mammals, birds, fish and amphibians and shows generic features in these different species [36]–[40]. A recent study analysed the early *Hox* expression patterns in *Xenopus*, and this revealed temporally colinear initiation of expression of a sequence of *Hox* genes within a horseshoe-shaped domain of ventrolateral marginal zone mesoderm with the tips of the horseshoe facing dorsal at different stages during gastrulation and then sequential dorsalisation of each *Hox* expression zone corresponding with its translation into a stable AP pattern zone in axial mesoderm and the neural plate [40], [41]. This sequence reflects timed interactions between an early ventrolateral mesodermal *Hox* cascade and the Spemann organiser that are probably imperative for AP axis formation.

We set out to investigate whether early expression of *Hox* genes depends on the activity of XMeis3 and whether XMeis3 is involved in regulation of expression of these *Hox* genes in mesoderm during gastrulation. In order for *XMeis3* to be able to regulate *Hox* expression in mesoderm, it and *Hox* genes need to be co-expressed there. We performed whole mount *in situ* hybridisation to study the detailed early expression of *XMeis3* and compared it to the early expression patterns of *Hoxd1*, *Hoxb4*, and *Hoxc6* and found significant co-expression in lateral regions of marginal zone mesoderm, early during gastrulation. This is the first time that Xmeis3 expression has been reported in gastrula mesoderm. To gain further insight into the early functions of *XMeis3*, we followed a gain- and a loss-of-function strategy. In the gain-of-function strategy synthetic *XMeis3* mRNA was microinjected into early blastomeres and expression of *Hox* genes was studied. These experiments showed that ectopic expression of *XMeis3* during gastrulation is capable of inducing expression of the *Hox* genes assayed in mesoderm as well as in ectoderm. In the loss-of-function strategy we made use of an antisense morpholino oligonucleotide (reviewed in [42] and references therein) to inhibit the translation of *XMeis3* mRNA (MO*^XMeis3^*). Injection of MO*^XMeis3^* leads to a reduction in expression of *Hoxd1*, *Hoxb4*, and *Hoxc6* in mesoderm and ectoderm during gastrulation, and to severe patterning defects. Finally we show synergy between Hoxd1 and XMeis3 and show that the mesodermal expression of *Hoxd1* during early gastrulation is already dependent on XMeis3 mediated autoregulation.

## Results

### The expression of *XMeis3* overlaps with *Hox* gene expression in mesoderm

**Figure 1:**
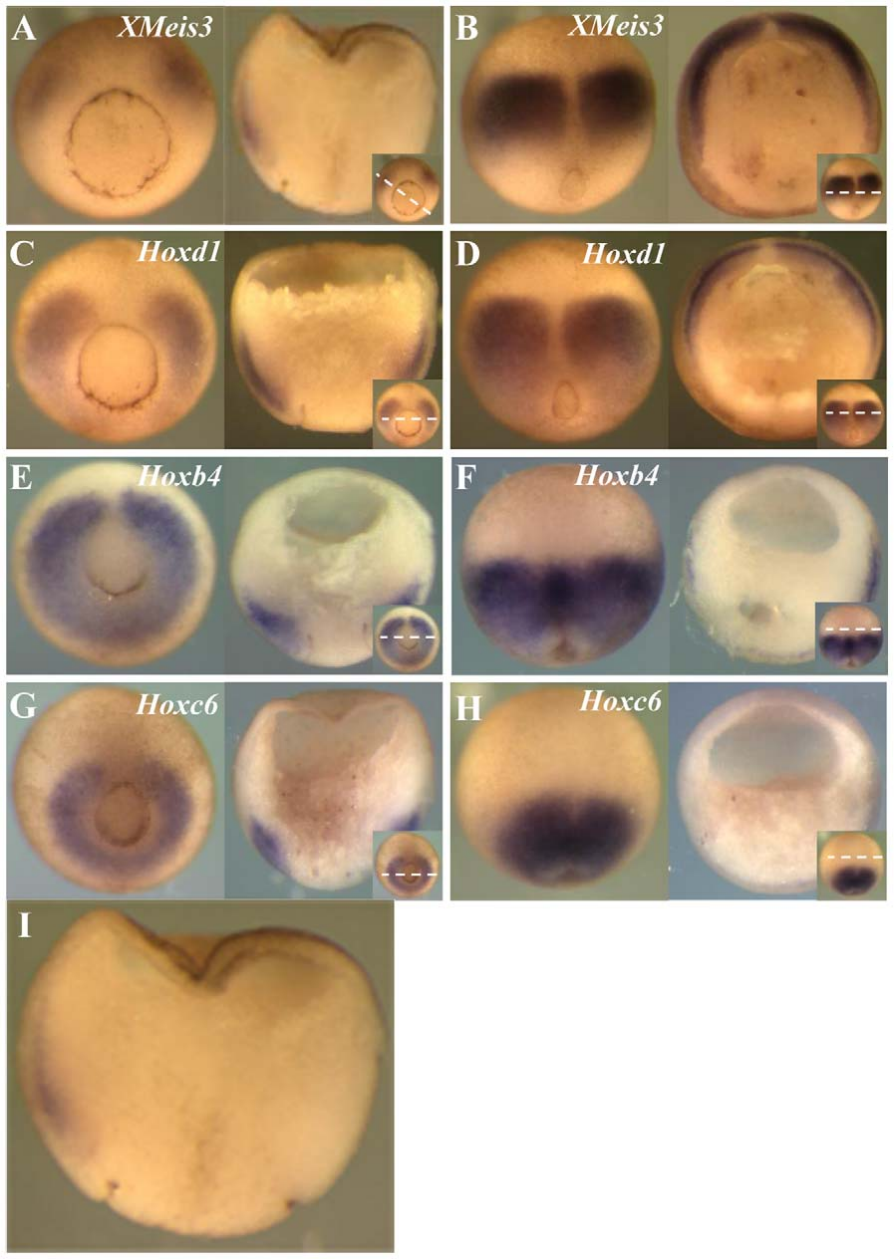
Expression of *XMeis3*, *Hoxd1*, *Hoxb4*, and *Hoxc6* during gastrulation. Embryos were analysed by whole-mount *in situ* hybridisation for expression of *XMeis3* (**A** and **B**), *Hoxd1* (**C** and **D**), *Hoxb4* (**E** and **F**), and *Hoxc6* (**G** and **H**). Whole mounts are shown on the left side of each panel, sections of these embryos are shown on the right side of each panel, in the inset, on the bottom right corner of every panel, the dotted line indicates the plane of sectioning. Spemann's organiser is clearly visible in Figs 1A,C,E, as the gap in the Hox or Meis expression domain, facing up in the left hand panels . Embryos shown are at stage 11, vegetal views with dorsal up (**A**, **C**, **E**, and **G**) and at stage 13, dorsal views with anterior up (**B**, **D**, **F**, and **H**). *XMeis3* expression overlaps with dorsolateral expression of *Hoxd1*, *Hoxb4*, and *Hoxc6* in mesoderm at stage 11 (**A**, **C**, **E**, and **G**). *XMeis3* expression in ectoderm at stage 13 overlaps with expression of *Hoxd1* but not with expression of *Hoxb4* and *Hoxc6* (**B**, **D**, **F**, and **H**). At stage 11, Hox and Meis expression is limited by a sharp boundary, running parallel to the outside of the embryo. This boundary is Brachy's cleft, the boundary between involuted mesoderm and external ectoderm Brachy's cleft runs from the blastopore to the upper limit of the involuted mesoderm (and is actually visible as a cleft in the upper part of the right panel of Fig 1C). All early Hox expression is known to be inside this cleft at this stage (mesodermal, not ectodermal) and thus marks the position of the cleft. The early XMeis3 expression shows the same pattern. It is mesodermal. At a later stage (st.13, Fig 1B), [40] XMeis 3 expression is also outside Brachy's cleft (ectodermal).

To determine whether *XMeis3* is co-expressed with *Hox* genes in the mesoderm of gastrula embryos, whole mount *in situ* hybridisation was performed for *XMeis3*, *Hoxd1*, *Hoxb4*, and *Hoxc6* (Fig. 1). Expression of *XMeis3* is initiated in a horseshoe-shaped domain in ventrolateral marginal zone mesoderm of the early gastrula (st. 10.5) (the tips of the horseshoe face dorsal). By stage 11, expression is lost in the ventralmost tissue, resulting in two lateral expression domains, one on either side of the organiser in mesoderm of early gastrula stage embryos (Fig. 1A). Expression thus becomes localised to mesoderm lateral to the midline and to a very low extent also possibly to the overlying ectoderm (Fig. 1A). Expression later, at the beginning of neurulation (st.13) is primarily in neurectoderm, as has been reported previously [52] but there is also remaining expression in dorsolateral mesoderm (Fig. 1B). Early expression of *Hoxd1*, *Hoxb4*, and *Hoxc6* is initiated in ventrolateral mesoderm and each of these genes follows a similar spatiotemporal expression sequence but with specific timing [40]. During early phases of gastrulation mesodermal expression of *Hoxd1* (Fig. 1C), *Hoxb4* (Fig. 1E), and *Hoxc6* (Fig. 1G) overlaps with expression of *XMeis3* in the dorsolateral domains of these *Hox* genes (compare Fig. 1A to 1C, 1E, and 1G). At the end of gastrulation, the overlap between mesodermal expression of *Hoxd1* (Fig. 1D) and *XMeis3* (Fig. 1B) in mesoderm is maintained, and the newly initiated expression of both genes in the neurectoderm also overlaps. At the same time, the more posteriorly expressed *Hoxb4* (Fig. 1F) and *Hoxc6* (Fig. 1H) only partially overlap *XMeis3* expression (Fig. 1B) in involuted mesoderm. *Hoxb4* expression also partially overlaps expression of *XMeis3* in overlying ectoderm (compare Fig. 1F to 1B). These results show that there is indeed an overlap in expression of *XMeis3* and of early *Hox* genes in mesoderm during gastrulation, and that expression of *XMeis3* also overlaps with *Hoxd1*, and to some degree *Hoxb4*, in neurectoderm.

### *XMeis3* gain-of-function upregulates *Hox* gene expression in mesoderm and ectoderm

**Figure 2:**
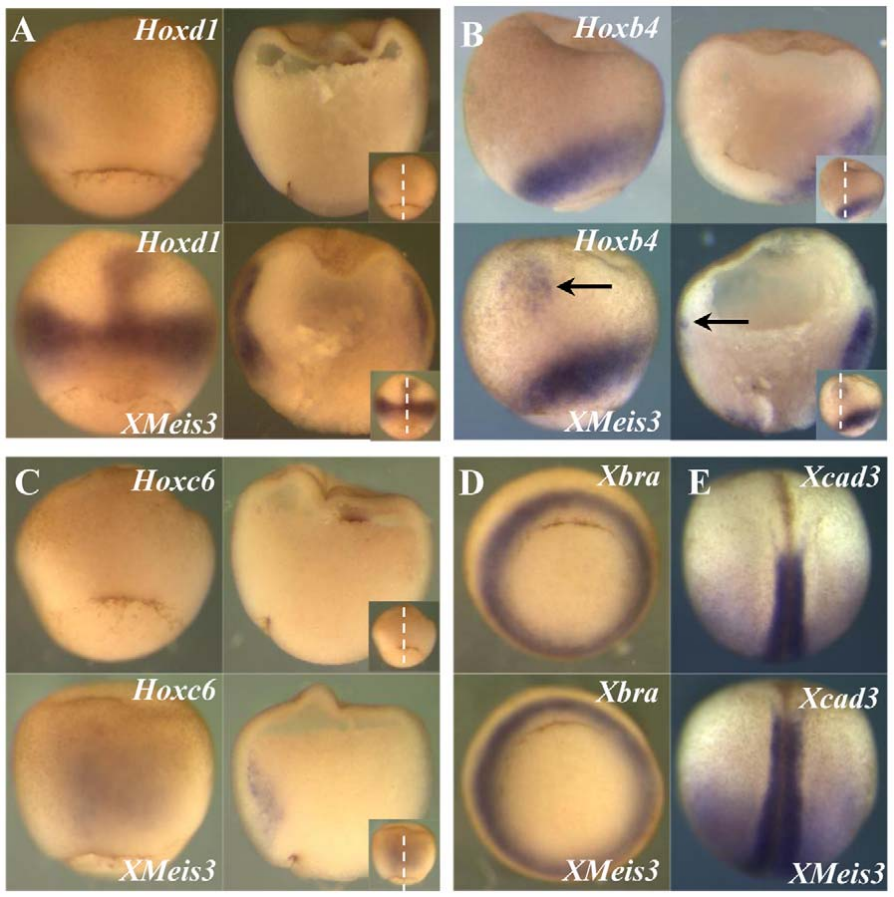
XMeis3 gain-of-function. Embryos were injected into the animal hemisphere at the one-cell stage with 2 ng synthetic mRNA containing the full-length coding region of *XMeis3*, and analysed by whole-mount *in situ* hybridisation. In each panel, control embryos are shown on top, the *XMeis3* injected embryos are shown on the bottom. Each letter indicates at least a pair of images: one embryo injected with XMeis3 mRNA (experimental, labeled XMeis3), one not (control, unlabelled). The label above on each image indicates the gene being assayed; the label below, if present, indicates XMeis3 injection (or no injection, if not present). For D and E, there are only images of intact embryos processed for whole mount in situ hybridization. For **A**, **B**, and **C**, two whole mounts are shown on the left hand side, and sections of these embryos are shown on the right hand side of each panel. Each of these letters thus represents four images. The plane of sectioning is depicted by the dotted line in the insets of **A**, **B**, and **C**. (**A**) Expression of *Hoxd1*, whole mounts are shown in dorsal view, with anterior to the top, at stage 10.5. Lateral expression of *Hoxd1* in injected embryos is stronger and in a broader domain, the gap in expression on the dorsal mesoderm is closed and a streak of expression in dorsal mesoderm is observed. (**B**) Expression of *Hoxb4*, whole mounts are shown in lateral view, with dorsal to the left, at stage 11. Lateral expression of *Hoxb4* is not affected by injection of *XMeis3*, the black arrow points to a patch of ectopic expression in ectoderm. This is joined to the mesodermal expression domain by a very faint streak of expression. (**C**) Expression of *Hoxc6*, whole mounts are shown in dorsal view, with anterior to the top, at stage 10.5. Injected embryos show extensive early ectopic expression of *Hoxc6* in dorsal mesoderm, prior to initiation of endogenous expression of *Hoxc6*. Please note that this early induced expression of the Hox genes is clearly mesodermal (internal to Brachy's cleft) and not ectodermal (surface expression) (**D**) Expression of *Xbra*, embryos at stage 10.5 are shown in vegetal view with dorsal to the top. No change can be observed in the expression of the mesodermal marker *Xbra* as a result of injection of *XMeis3*. (**E**) Expression of *Xcad3*, embryos at stage 17 are shown in dorsal view with anterior to the top. The anterior expression boundary of the posterior marker *Xcad3* is shifted to a more anterior position following injection of *XMeis3*. Spemann's organizer is indicated by the crescent stripe, bottom centre, in the upper left panels of Figs. 2A and 2C.

To investigate whether *XMeis3* is capable of contributing to the regulation of *Hox* gene expression, 2 ng of synthetic mRNA containing the full-length coding region of *XMeis3* was injected into the animal pole of embryos at the one-cell stage. The amount of 2 ng was chosen because this was shown to lead to posteriorisation of injected embryos [26]. The effects on expression of *Hoxd1*, *Hoxb4*, *Hoxc6*, *Xbra*, and the posterior marker *Xcad3* in gastrula stages were assayed by *in situ* hybridisation (Fig. 2). The ectopic expression of *Hoxd1* (Fig. 2A) in injected embryos is remarkable because it is found in the region harbouring the Spemann organiser, tissue that normally does not express *Hox* genes. The horseshoe-shaped domain of expression is also expanded and expression levels appear to be enhanced. Furthermore expression can be found in ectoderm of the animal cap and in the mesoderm underlying it, in the form of a streak of expression in contact with the expanded ring of expression around the blastopore (Fig 2A). *Hoxb4* also shows ectopic expression in animal cap ectoderm and expansion of the endogenous expression domain (Fig. 2B), but no closure of the dorsal expression gap neither in organiser mesoderm nor in overlying ectoderm can be observed. Interestingly, induced expression of *Hoxc6* can already be found in dorsal mesoderm at stage 10.25 (Fig. 2C), significantly earlier than its endogenous initiation of expression (st11) and like ectopic *Hoxd1* expression, this occurs in dorsal mesoderm. In later stages an expansion of the endogenous horseshoe-shaped expression domain is also found (data not shown). Expression of the mesodermal marker *Xbra* appears unaltered in injected embryos (Fig. 2D), suggesting that changes in *Hox* expression domains are not due to changes in induction of mesoderm, but rather to its patterning. The previously described posteriorising effect of *XMeis3* on neurectoderm is confirmed by anterior expansion of expression of the posterior marker *Xcad3* (Fig. 2E).

### *XMeis3* loss-of-function downregulates expression of *Hox* genes and arrests gastrulation

**Figure 3:**
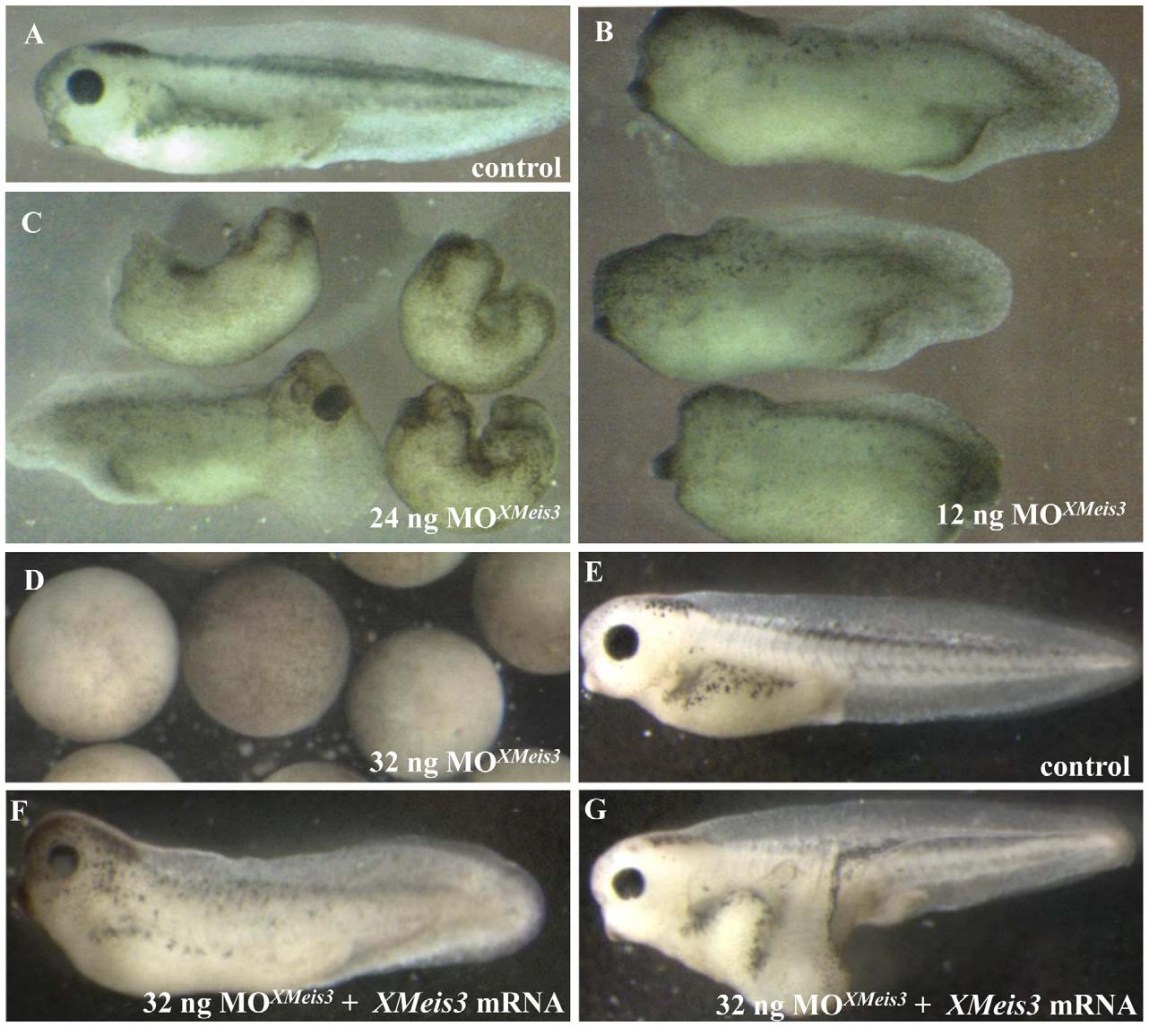
Effects of *XMeis3* MO loss-of-function on embryonic development and the rescue of MO*XMeis3*. Embryos at the one-cell stage were injected into the animal hemisphere with MO*XMeis3* in amounts of 12 ng (**B**), 24 ng (**C**), and 36 ng (**D**), and allowed to develop until the control embryos (**A**) reached tadpole stages. This treatment disturbs development of the embryonic axis. At the highest concentration, the embryo is blocked during gastrulation (fig. 3D) and then disintegrates to a mass of dissociated cells contained within the vitelline membrane (not shown). The specificity of MO*XMeis3* is shown by the rescue with *XMeis3* synthetic mRNA. Embryos were injected with 32 ng of MO*XMeis3* and 125 pg synthetic mRNA for *XMeis3* and allowed to develop until the control embryos reached the tad pole stage (**E**), In the majority of the embryos a large part of the axis was rescued (**F**), in a small number of embryos the phenotype could even be reversed, not only is the axis fully rescued but the embryo shown in (**G**) even possesses additional trunk structures as was revealed by the presence of somites in the axis outgrowth (not shown). The most extreme MO treatment thus produced a gastrulation block. Other treated embryos were allowed to develop to comparable stages (¬ 40–45) as shown by development of stage specific structures, for example the cement gland (seen best in Figs 3A, B, C, F G as the black spot at the lower front end of each embryo. Front ends are left in 3A, B, E, F, G. Various directions in 3C.

To determine whether *XMeis3* function is necessary for initiation and/or establishment of *Hoxd1*, *Hoxb4*, and *Hoxc6* expression, an antisense morpholino oligonucleotide directed against *XMeis3* mRNA (MO*^XMeis3^*) was injected into the animal hemisphere of embryos at the one-cell stage. *XMeis3* loss-of-function leads to a loss of trunk structures and defects in axis specification, in a concentration dependent manner. When 12 ng MO*^XMeis3^* was injected a loss of trunk structures and defects in head development and tail formation can be observed, while the anteriormost structure, the cement gland, remains present (Fig. 3B). When 24 ng MO*^XMeis3^* was injected, an enlargement of the cement gland was visible accompanied by a stronger loss of trunk structures (Fig. 3C) In half the injected embryos *spina bifida's* are observed, suggesting that the embryos suffer from gastrulation problems. When 32 ng or more MO*^XMeis3^* were injected, the embryos arrested during gastrulation at stage 11 (Fig. 3D). Embryos injected with this high dose of MO*^XMeis3^* appear unaffected and posses normal looking blastopores until the moment of arrest. This is unlike what would be expected if the arrest was caused by toxicity of an injected agent, this would generally generate a much larger spread in stages at which embryos die or arrest, accompanied by irregular formation of the blastopore. Removal of the vitelline membrane revealed that cells have lost cell-cell contact, but appear round and intact (not shown). This suggests that the observed effect is the result of a strong knockdown of *XMeis3* function and not an aspecific effect of MO*^XMeis3^*. Injection of the same amount of a control morpholino (MO^contr^), in sequence unrelated to MO*^XMeis3^*, has no outward effects on embryos (data not shown). These findings support the idea that the gastrulation arrest phenotype is a true result of XMeis3 loss-of-function and that XMeis3 is required for patterning (a part of) the primary axis in *Xenopus* embryos. Actually, this result is perhaps not so surprising because: a recent result shows that EMT timing during internalisation of mesoderm into the gastrula is regulated (delayed) by hox genes [43] and because we present evidence (below) that the important function of Meis3 in the gastrula is to mediate mesodermal autoregulation of *Hox* genes.

To further test the specificity of the MO*^XMeis3^*, 125 pg of synthetic *XMeis3* mRNA, lacking most of the sequence that the MO*^XMeis3^* is complementary to, was co-injected with 32 ng MO*^XMeis3^* into the animal hemisphere of embryos at the one-cell stage (Fig. 3F). The exogenous *XMeis3* was able to largely rescue the MO*^XMeis3^* phenotype (compare Fig. 3D to 3E, and 3F). In a small number of the co-injected embryos a full recovery of the axis can be observed, sometimes accompanied by a secondary axial outgrowth out of the primary axis, containing somites (Fig. 3G).

**Figure 4:**
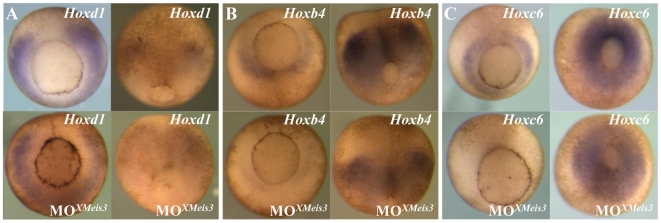
XMeis3 loss-of-function. Embryos were injected at the one-cell stage with 16 ng of the MO*XMeis3*, and analysed by whole mount *in situ* hybridisation at stage 10.5/11, shown on the left side of each panel, and at stage 12, shown at the right side of each panel. Injected embryos are shown at the bottom of each panel, untreated embryos are shown on top. Shown are vegetal views with dorsal to the top. Expression of *Hoxd1* (**A**), *Hoxb4* (**B**), and *Hoxc6* (**C**) is downregulated in mesoderm of injected embryos at early gastrula stages. A reduction in neurectodermal expression of the three *Hox* genes studied, is also observed in injected embryos at stage 12.

The effect of XMeis3 loss-of-function on *Hox* expression was studied by injecting 16 ng MO*^XMeis3^* into the animal hemisphere of embryos at the one-cell stage followed by *in situ* hybridisation at appropriate stages. To be able to analyse marker expression in late gastrula stage embryos, the arrest in gastrulation, observed after injection of a high amount of MO*^XMeis3^*, was avoided, by the injection of 16 ng. The XMeis3 loss-of-function leads to downregulation of expression of *Hoxd1* (Fig. 4A), *Hoxb4* (Fig. 4B), and *Hoxc6* (Fig. 4C), early in mesoderm and later in neurectoderm. This led to our conclusion that XMeis3 is necessary for *Hox* gene expression in marginal zone mesoderm, and neural plate ectoderm.

### Synergy between *Hoxd1* and *XMeis3*

Autoregulation is known to occur among labial type *Hox* genes in murine hindbrain neurectoderm [21], [44], in endoderm of *Drosophila* embryos [17], [25], and in *C. elegans* [23] For a number of these cases it has been shown that this autoregulation is dependent on a Pbx/Hox bipartite binding site in the *Hox* promoters [17], [21], [23], [25]

**Figure 5:**
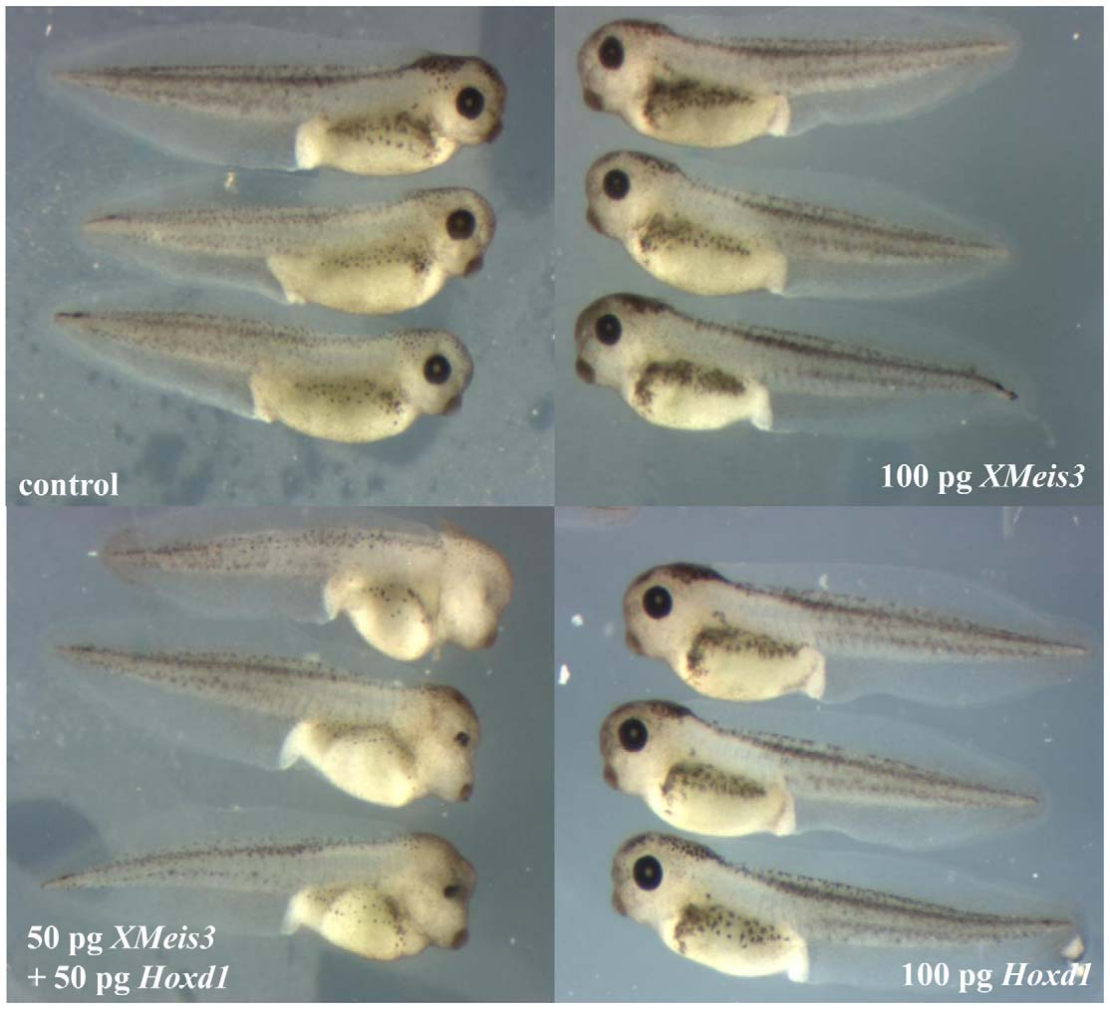
Synergistic effect between *Hoxd1* and *XMeis3* in ectopic expression. Embryos at the one-cell stage were injected into the animal hemisphere with either 100 pg *Hoxd1* mRNA, 100 pg *Xmeis3* mRNA, or 50 pg of both mRNA's. A single injection of 100 pg of either factor is not sufficient to induce a phenotypic effect. The combination of half the amount of *Hoxd1* and *XMeis3*, results in posteriorisation, shown by a clear reduction of eye formation, and an anterior shift of the eye.

Because nuclear localisation of Pbx family members is dependent on the action of Meis family members and because XMeis3 loss-of-function led to a significant downregulation of *Hoxd1* expression in mesoderm and ectoderm, we suspected that *XMeis3* might be involved in Hoxd1 autoregulation. To test our idea that XMeis3 may mediate autoregulation of labial type *Hox* genes in *Xenopus* development, we co-injected relatively small amounts of synthetic mRNA for *XMeis3* and *Hoxd1* and also injected them separately using double the amount of mRNA. Small amounts of mRNA were used to be able to observe compound phenotypes in co-injected embryos. If a strong effect was generated in embryos injected with only a single messenger this would not have been possible. The embryos injected with only a single synthetic messenger show little or no phenotypic effect, while co-injected embryos show a significant retardation in head development (Fig. 5). This points towards a synergistic relation between Hoxd1 and XMeis3.

**Figure 6:**
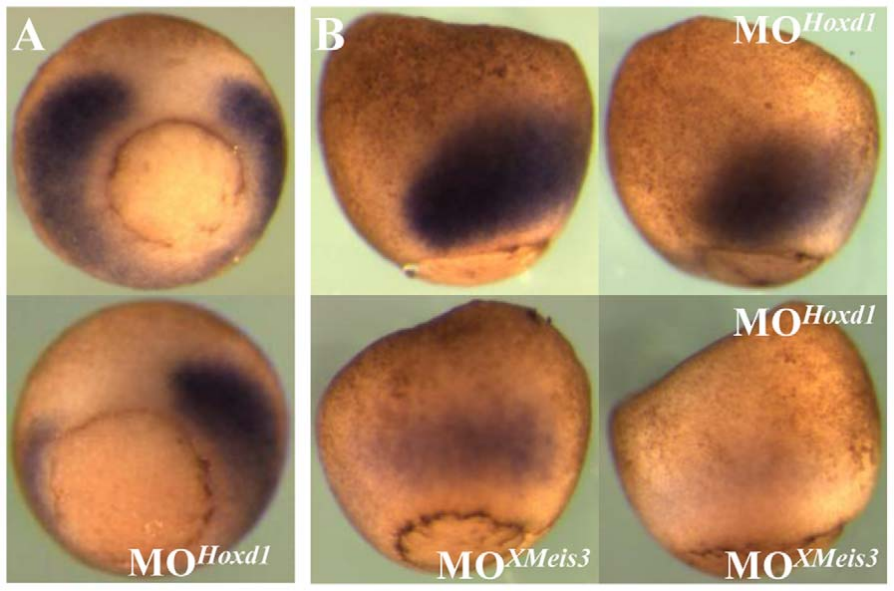
Synergistic effects in loss-of-function of Hoxd1 and XMeis3. (**A**) Embryos were injected with 362 ng of MO*Hoxd1* into the lateral marginal zone on the left side of embryos, rendering the un-injected side an internal control. Embryos were allowed to develop until control stage 11 and assayed by *in situ* hybridisation for expression of *Hoxd1*. Embryos are shown in vegetal view, with dorsal up. Expression of *Hoxd1* is reduced on the left side of injected embryos (shown on the bottom of the panel). (**B**) To investigate whether there is synergy between Hoxd1 and XMeis3, 16 ng MO*XMeis3* and 16 ng MO*Hoxd1* were injected, together and separately, into the animal hemisphere of one-cell stage embryos. The embryos were harvested at st 11 and assayed for expression of *Hoxd1* by *in situ* hybridisation. Embryos are shown in lateral view, with dorsal to the left. Injection of either MO*Hoxd1* or MO*XMeis3* separately leads to a reduction in the early mesodermal expression of *Hoxd1*. Their co-injection leads to a further reduction in early mesodermal *Hoxd1* expression as compared to injection of either MO*XMeis3* or MO*Hoxd1* separately. This suggests that Hoxd1 and XMeis3 work synergistically in mediating establishment of *Hoxd1* expression in mesoderm during early gastrula stages.

To further test this synergy, and to test whether XMeis3-mediated Hoxd1 autoregulation is involved in the establishment of *Hoxd1* expression, we wished to investigate the necessity of Hoxd1 for maintaining *Hoxd1* expression in mesoderm. If XMeis3 activity is needed in early gastrula mesoderm to enhance or alter the function of Hoxd1, then Hoxd1 loss-of-function should generate the same effect on *Hoxd1* expression as XMeis3 loss-of-function. To test whether this is the case, 32 ng MO*^Hoxd1^* [53] was injected into the equatorial region of the 2 blastomeres making up the presumptive left side of 4-cell stage embryos. The other half of the embryos served as an internal control. This results in a downregulation of expression of *Hoxd1* in mesoderm on the injected side (Fig. 6A). This finding extends our recent investigation of the effect of MO knockdown of labial Hox genes on neurectodermal Hox gene expression [53]. To further test whether establishment of expression of *Hoxd1* needs both Hoxd1 and XMeis3, sub optimal amounts of morpholinos against both messengers were co-injected and injected separately. Embryos were harvested at stage 11 and assayed for *Hoxd1* expression (Fig 6B). Sub optimal morpholino amounts were used to allow different levels of reduction in *Hoxd1* expression, thus allowing possible synergistic effects to be observed. A downregulation of *Hoxd1* expression in embryos injected with a single morpholino and a strong additional reduction by injection of both morpholinos is visible (Fig. 6B). This suggests that there is indeed a synergistic effect of Hoxd1 and XMeis3 on establishment of *Hoxd1* expression in marginal zone mesoderm during gastrulation.

## Discussion

### *XMeis3* expression overlaps early *Hox* expression

Much effort has been put into finding out details about the relation between *Hox* proteins and their cofactors Pbx/Exd and Meis/Hth. Although much has been accomplished, many questions remain. In *Xenopus* embryos, it has been shown that *XMeis3* has a function in hindbrain patterning [26]–[28], these results are corroborated by recent reports concerned with Meis function in hindbrain formation in zebrafish embryos [29]–[31]. We show here that *XMeis3* is expressed in marginal zone mesoderm significantly earlier than previously described [26], [28]. We went on to show that an overlap is found between expression of *XMeis3* and of early *Hox* genes in ventral and lateral and dorsolateral mesoderm during gastrulation. At st. 11, the overlap is restricted to dorsolateral mesoderm. This co-localisation with early *Hox* genes is compatible with a role for *XMeis3* in the regulation of *Hox* gene expression in mesoderm during the early phases of gastrulation.

### Ectopic *XMeis3* enhances *Hox* expression in mesoderm

By gain-of-function experiments we showed that ectopic *XMeis3* is capable of inducing expression of *Hoxd1*, *Hoxb4*, and *Hoxc6*, expanding the endogenous expression domains of these genes in early mesoderm, and ectopically initiating expression in dorsal mesoderm. Interestingly, this induction of *Hox* expression by ectopic *XMeis3* can only be found as expansions of the endogenous expression domains or in streaks of expression still in contact with the expanded endogenous domains of expression. This is most obvious for ectopic expression of *Hoxd1* in dorsal mesoderm, expanding into more animally located mesoderm and ectoderm. This suggests that ectopic *XMeis3* only enhances the expression of the assayed *Hox* genes, requiring factors already present in their endogenous *Hox* expression domains rather than inducing expression *de novo*. We suspect that the endogenous factors required are the *Hox* proteins themselves. These patterns are consistent with our idea (below) that *XMeis3* enhances *Hox* autoregulation in mesoderm of *Xenopus* embryos.

### *XMeis3* is necessary for *Hox* expression in mesoderm and ectoderm

The injection of MO*^XMeis3^* led to a downregulation of mesodermal expression of all three *Hox* genes assayed. For *Hoxd1* and *Hoxb4* this held true for mesoderm and ectoderm, in the case of *Hoxc6*, mesodermal expression partially recovers during later phases of gastrulation, but ectodermal expression could not be observed. This indicates that *XMeis3* protein is necessary, in ventral and lateral mesoderm and in neurectoderm during gastrulation, for proper establishment and maintenance of *Hox* expression.

XMeis3 loss-of-function using small amounts of MO*^XMeis3^* already led to a strong phenotype, indicating the necessity of XMeis3 function in anteroposterior patterning. This phenotype corroborates the results of Dibner and co-workers [27]. The sudden arrest in gastrulation at stage 11, caused by injecting a high amount of MO*^XMeis3^* is very striking. We show by coinjecting a limited amount of *XMeis3* mRNA that the observed effect is not aspecific. We note that there is published evidence that Hox genes regulate cell movement and EMT's during gastrulation [43] and suspect that this *XMeis3* effect is connected with this. This is possibly due to an effect on Hox/Meis synergy: See below. The phenotype observed after injection of less morpholino, namely loss of trunk structures, head defects, and retarded tail formation described in this report and by Dibner and co-workers [27], is therefore most likely a result of reduced XMeis3 function, not a complete loss of function. We cannot be certain that the phenotype caused by injection of 32 ng MO*^XMeis3^* represents the complete loss-of-function phenotype, but it suggests the need for XMeis3 in two processes during early development: the progression of gastrulation and *Hox* expression and patterning in the early mesoderm and hindbrain.

### Synergy between *Hoxd1* and *XMeis3*

We suspected that Meis3 is important for Hox expression because it mediates Hox autoregulation so we tested whether Hoxd1 and Meis3 synergise in early gastrula mesoderm. The synergistic effects we have observed in the gain-of-function experiment by injection of synthetic *XMeis3* and *Hoxd1* mRNA together show that these two factors, when co-expressed can indeed generate a phenotype that cannot be accomplished by injecting double the amount of either factor separately. These results recall the findings of Vlachakis and co-workers [29], who have shown that in zebrafish embryos, Meis3, Pbx4, and Hoxb1 synergise to promote hindbrain fate. Combined Hoxd1 and XMeis3 loss-of-function also indicates synergy; while sub optimal amounts of either morpholino against *Hoxd1* or *XMeis3* led to a reduction of *Hoxd1* expression, the combination led to a much stronger reduction. This adds to the evidence for a synergistic relation between Hoxd1 and XMeis3. Taken together our results show that *XMeis3* is necessary in marginal zone mesoderm to establish the early expression of *Hox* genes. This *XMeis3*-mediated mesodermal *Hox* cascade is of vital importance for axis formation and AP patterning.

### Autoregulation by *Hoxd1* is necessary for establishment of its expression in marginal zone mesoderm

Autoregulation dependent on Pbx has been shown for Hox paralog group 1 and 4 members in neurectoderm [17], [21]–[24], [44]. This suggests that the the regulation of *Hox* expression by XMeis3 that we have demonstrated could take place at the level of Hox autoregulation. Indeed, injection of MO*^Hoxd1^* led to a reduction in *Hoxd1* mRNA expression. The expectation is that this is the result of a reduction in *Hoxd1* translation, leading to a reduced amount of Hoxd1 protein and we suspect that this causes a reduction in availability of *Hoxd1* mRNA because of autoregulation. This suggests that Hoxd1 autoregulation is an essential step in the establishment (but not initiation), and not only the maintenance (as in neurectoderm), of *Hoxd1* expression in mesoderm during gastrulation in *Xenopus* embryos. We do not yet know whether this autoregulation is direct or indirect and have no evidence as to the mechanism. However, the involvement of *Meis3* suggests that it is by the known mechanism [17], [21]–[25], [44]. The observed reduction of *Hoxd1* expression could also be explained if binding of MO*^Hoxd1^* to mRNA led directly to destabilisation of the *Hoxd1* messenger, however this effect has, to our knowledge, not been reported and our findings (above) of the necessity of *Meis* for mesodermal Hox expression and for synergy between *Hoxd1* and *Meis3* also point strongly to autoregulation via the known *Meis* dependent mechanism [17], [21]–[25], [44]. The necessity for *Hoxd1* autoregulation in mesoderm is a remarkable discovery considering that vertebrate Hox autoregulation has previously only been shown in the hindbrain We note that *Hoxd1* loss-of-function is clearly not fully, if at all, rescued by the other labial type Hox genes; : *Hoxa1* and *Hoxb1* that are normally co-expressed during gastrulation. Either *Hoxa1* and *Hoxb1* are not capable of inducing the expression of *Hoxd1*, which seems unlikely taking into account the redundant functions of these paralog group members (reviewed in [45] and references therein), or expression of *Hoxa1* and *Hoxb1* is reduced or prevented by *Hoxd1* loss-of-function. This second idea would suggest the necessity of *Hoxd1* to induce the two other labial homologous (which are expressed slightly later) during gastrulation in *Xenopus* embryos. Additional experiments are needed to distinguish between the two possibilities but whatever the outcome, this finding sheds new light on the initiation and establishment of expression of the early gastrula *Hox* cascade. Obviously, auto and cross regulation can not be involved in initiating the very first expression of Hox genes. We conclude that autoregulation is involved only in the establishment and maintenance phases of Hox expression and not initiation. In fact, we have evidence that Hox expression in the Xenopus gastrula is initiated by Wnt8, which directly induces expression of Hoxd1 and of its paralogues but not of other Hox genes [54].

### Concluding Remarks

Our investigations shed new light on the roles of Meis3 and of Hox genes in early embryonic development and axial patterning. We made four main findings which relate to the role of the early gastrula non organiser mesoderm which has recently been shown to be very important in early embryonic patterning [40], [41]. This early mesoderm is important because it is the first embryonic tissue to express *Hox* genes. It has a temporally collinear sequence of *Hox* gene expression that is used to ste up the spatially collinear *Hox* sequence in the later embryo's axial pattern by time- space translation [40], [41] We show here that *Xmeis3* and *Meis-Hox* synergy are needed for setting up this early mesodermal *Hox* sequence

1/ We showed for the first time that *Meis3* starts to be expressed earlier in the early Xenopus embryo than preiously reported: in the non organiser mesoderm at the early gastrula stage St 10.5 rather than the early neurula stage, after gastrulation. This early mesodermal *Meis* expression overlaps with the early mesodermal expression of the *Hox* genes.
2/ We showed for the first time that artificial ectopic expression of *Meis3* causes ectopic expression of *Hox* genes in the early gastrula non organiser mesoderm as well as in embryonic neurectoderm. This ectopic expression occurs only in tissue that is in contact with non organiser mesoderm expressing the *Hox* gene in question or another *Hox* gene, indicating the need for additional endogenous factors for ectopic expression. We speculate that these may be the Hox proteins themselves. This finding constituted our first piece of evidence suggesting that *Meis3* may be needed for early gastrula *Hox* expression.
3/ We showed for the first time that *Meis3* loss of function via antisense oligonucleotide morpholinos blocks or downregulates *Hox* gene expression in early gastrula non organiser mesoderm. This is evidence that mesodermal *Meis* is indeed needed for mesodermal *Hox* expression.
4/ We showed for the first time that endogenous and ectopic *Meis3* and *Hoxd1* can and do synergise to induce *Hoxd1* expression in early gastrula mesoderm. This is evidence that synergy between Meis and Hox mediates mesodermal expression of at least one Hox gene. We believe that this reveals a detail of how *Meis3* regulates *Hoxd1* expression.

## Materials and Methods

### *Xenopus* embryos and microinjections

Pigmented *Xenopus laevis* embryos were obtained by *in vitro* fertilisation, and after dejelling in a 2% cysteine solution (pH 8.0), cultured in 0.1× Marc's Modified Ringers's (MMR) [46] containing 50 µg/ml gentamycin at 14–21°C. Embryos were injected in 1× MMR+4% ficoll and afterwards transferred to 1× MMR+1% Ficoll, and cultured in this medium for 1 to 7 hours, after which they were transferred and to 0.1× MMR in which they were cultured until harvesting. Staging of the embryos was performed according to Nieuwkoop and Faber [47]. Embryos at the one-cell stage were injected into the animal pole with synthetic mRNA dissolved in water. The synthetic capped mRNA was made using the Ambion mMessage mMachine Kit with CS2-*XMeis3*, or CS2-*Hoxd1*, linearised with *Not*I, as template. CS2-*XMeis3* was constructed by cloning the full-length coding region of *XMeis3*, obtained by PCR using stage 15 cDNA as template and the following primers: f: 5′-gcgggatccatggcacaaaggtatgatgag, r: 5′–cgcctcgagcatgtagtgccactgcccctcc, containing an *BamH*I or a *Xho*I restriction site respectively, in the CS2+ vector [48] using the restriction sites in the primers. CS2-Hoxd-1 contains the complete coding sequence of *XHoxd1* in CS2+, kindly provided by W. Van den Akker.

MO*^XMeis3^* Gene Tools, LLC, (directed against the XMeis3 mRNA's 5′ region) has the sequence: 5′-cctttgtgccattccgagttgggtc, and was injected in amounts of 8 to 36 ng. in a concentration of 8 ng/nl. MO^contr^, supplied by Gene Tools, LLC, has the licence: 5′-cctcttacctcagttacaatttata and was injected using the same amounts and concentrations as MO*^XMeis3^*.

### Whole mount *in situ* hybridisation and antisense probes

Whole mount *in situ* hybridisations were performed according to Harland (1991), with minor modifications. The antisense RNA probes were generated by run off *in vitro* translation using DIG RNA labelling mix (Roche), and T7 or Sp6 RNA polymerase (Promega). The probes were generated using the following templates: *Hoxd1*: [49], *Hoxb4*: a 708 bp fragment containing the complete *Hoxb-4* ORF cloned in pGEMTE, *Hoxc6*: a 998 bp *Hoxc-6* fragment in pGEM1 containing a part of the homeodomain and extending into the 3′ UTR, *Xcad3*: [50]; *Xbra*: pSP73Xbra [51].

### Ethics

Our work uses early Xenopus embryos. It is carried out according to national and EU guidelines and regulations. The animal work is covered by a ‘DEC’ licence, No. 513, covering ethical aspects, issued by the University of Leiden's Animal Experimentation Committee (Dier Experimenten Commissie) to A.J.Durston. The molecular work is covered by an existing COGEM licence No. GGO 02-055, issued to H.P.Spaink. This work requires no other licence.
